# Oral Baclofen Withdrawal Resulting in Hyperactive Delirium: A Case Report

**DOI:** 10.7759/cureus.14979

**Published:** 2021-05-12

**Authors:** Benjamin J Sanders, Ziad Ali

**Affiliations:** 1 Psychiatry, University of Kentucky, Bowling Green, USA

**Keywords:** oral, baclofen, delirium, psychosis, withdrawal

## Abstract

Baclofen is used in the treatment of muscle spasms by acting as an agonist GABA_B_ receptors. Limited case reports describing baclofen withdrawal-induced delirium have largely been demonstrated to occur upon cessation of intrathecal baclofen administration. Fewer reports have characterized the presentation from oral baclofen withdrawal. We describe a case of a patient, MM, who presented with hyperactive delirium with various, associated, behavioral, perceptual, cognitive, and movement disturbances following abrupt withdrawal of oral baclofen. Symptomatic management of occurring during the withdrawal state with antipsychotics and benzodiazepines has been reported previously. Similarly, MM’s perceptual and behavioral disturbances were managed with antipsychotics during her admission. However, addressing the underlying cause by reinstating and slowly tapering off baclofen has also been demonstrated to be an effective, targeted approach.

## Introduction

Baclofen is a synthetic chlorophenyl-butanoic acid derivative that is utilized for treatment of muscle spasms by acting as an agonist on GABAB receptors [1]. Abrupt withdrawal of baclofen has been found to demonstrate a range of adverse reactions including behavioral, perceptual, emotional, and movement disturbances [2-5]. We present a case demonstrating the evolving presentation of oral baclofen withdrawal to improve early recognition and expedition of appropriate management.

## Case presentation

MM is a 58-year-old female who presented to the emergency department (ED) on four separate occasions, all days apart from each other, with complaints of twitching in her upper and lower extremities, lower extremity weakness, and falls that had become increasingly worse over the past four days.

MM had a past psychiatric history significant for depression and past medical history significant for fibromyalgia and chronic back pain, which was managed with trazodone 100 mg nightly, baclofen 10 mg three times daily, as needed, oxycodone 10 mg every 8 hours, as needed, and zolpidem 10 mg nightly.

During her initial evaluation in the emergency room, MM demonstrated significant difficulty with concentration, tangential and disorganized thinking with occasional non-goal directed responses, impaired reasoning, “strange affect,” “bizarre behaviors,” with one provider reporting she was “talking out of her head.” Upon evaluation, a basic metabolic panel (BMP) found to be significant for an acute kidney injury with a blood urea nitrogen (BUN) of 36 and creatinine of 1.09 and mildly tachycardic and tachypneic. All other initial laboratory results, including complete blood count (CBC) and urinalysis, were found to be unremarkable. Urine drug screen was positive for oxycodone, to which she was prescribed. While in the ED, psychiatry was consulted for evaluation for disposition.

Upon evaluation, MM endorsed significant paranoia towards the nursing staff in the ED. She also demonstrated tangential thought processes with flight-of-ideas. She reported experiencing auditory hallucinations in the form of laughter and was observed responding to internal stimuli. She was found to have intact short- and long-term memory; however, she was alert and oriented only to self and location. She is also observed to have bilateral lower extremity weakness and abnormal upper extremity movements. She reported her mood as depressed, but demonstrated an expansive and incongruent affect.

Collateral information was obtained from MM’s son, who stated that he had also first noticed MM’s twitching roughly four days prior to her first ED visit. He also reported that around that same time, she had been intermittently appearing disoriented, demonstrating disorganized speech, and believed that MM had been experiencing auditory hallucinations. He reported that at her baseline, MM was coherent and oriented, and has never been observed responding to internal stimuli in the past.

At that time, it was recommended that MM be admitted to the hospital and worked up for medical/neurological causes for delirium. Initially, she was not continued on any of her home medications. On her first night after admission, MM required one PRN of 5 mg oral olanzapine and another 10 mg PRN of intramuscular ziprasidone for agitation. A head CT was ordered for acute mental status change and found only to demonstrate chronic small vessel disease. An EEG was also ordered and demonstrated mild to moderate diffuse encephalopathy. A urine culture found to be positive for E. coli; however, as she was asymptomatic, the primary medical team did not treat her asymptomatic bacturia.

On day 2 of MM’s hospital stay, she was observed to be alert and oriented to self and location with fluctuating orientation to location. MM continued to demonstrate involuntary twitches in the upper and lower extremities. At times, her speech was incoherent and disorganized. She was also occasionally observed responding to internal stimuli. Her affect was described as bizarre with inappropriate affect and incongruent to the stated mood of being “tired.” It was at this time, additional collateral was able to be obtained from MM’s daughter, who stated that MM had been taking up to triple her prescription of baclofen. Based on the information obtained from her daughter, as well as the previous information from her son and pharmacy records, it was believed that MM had run out of her baclofen about four days prior to her first presentation to the ED. This coincided with the first onset of her symptoms. It was also believed by some members of her care team that MM may have taken all of her prescribed baclofen and had recently run out in the days leading up to her presentation in the ED. For symptomatic management, psychiatry started MM on risperidone 1 mg twice daily for hallucinations and overnight agitation. Based on the information received from collateral, MM continued to be observed for delirium. However, as the treating teams were unsure whether MM’s presentation was due to baclofen overdose or withdrawal, MM continued to only receive symptomatic management early on during her stay.

Over the next two days, MM intermittently continued to be observed responding to internal stimuli and demonstrating bizarre behaviors, thought blocking, intermittent poverty of thought, impaired concentration, and fluctuating orientation. While the involuntary twitching in her extremities continued to decrease in frequency and intensity. Throughout her stay, MM continued to receive intravenous fluids. While her BUN and creatinine peaked on the third day of her admission at 56 and 2.84, respectively, with continued fluids her BUN and creatinine trended downwards with her last BUN and creatinine recorded at 34 and 1.17, respectively.

On day 6, MM was found to be much more calm and cooperative when seen by the psychiatry team on rounds. She was alert and oriented x3. Her thought processes were much more linear, goal-directed, and coherent. No bizarre behaviors or gestures were observed. While interviewing MM, she did report that she had been over-using her baclofen, but was unable to recall precisely how many pills she was taking daily. After discussion with the treating teams, based on MM’s presentation it was believed that delirium was primarily due to baclofen withdrawal that may have been exacerbated by an acute kidney injury. Psychiatry discussed the potential for future baclofen-induced delirium, especially if not taken as prescribed, with MM. She was discharged home with a plan to follow up with psychiatry and neurology on an outpatient basis.

## Discussion

The presentation of MM’s symptoms is consistent with previous case reports of baclofen withdrawal. The majority of published case reports of baclofen withdrawal delirium have occurred in the context of intrathecal baclofen administration; fewer case reports have been published examining the presentation of oral baclofen withdrawal delirium [2-6]. MM demonstrated musculoskeletal findings, such as abnormal, involuntary movements in her extremities throughout much of the early course of her hospitalization. Similar, intermittent, involuntary movements, and bilateral pupil dilation have also been described in various case reports regarding individuals presenting with baclofen withdrawal [2-6]. Similar to other accounts, MM demonstrated tachycardia when she presented to the ED; however, MM did not have similar characteristic vital signs, such as hypertension or hyperthermia that have also been reported [5].

Among MM’s most noteworthy and characteristic symptoms of baclofen withdrawal were her psychiatric symptoms. MM demonstrated significant impairments in her thought processes, which were noted to be tangential, disorganized, non-linear, and with flight of ideas, with one ED physician stating she was “talking out of her head.” Several case reports similarly describe patients in acute baclofen withdrawal who also demonstrated formal thought disorders, with one case report by Arnold et al. (1980) also describing the presentation of a patient in baclofen withdrawal who they described as “talking out of his head” [2-4]. Additionally, MM demonstrated impaired concentration, fluctuating orientation, perceptual disturbances in the form of auditory and visual hallucinations, paranoia, and sleep disturbances consistent with delirium due to baclofen withdrawal.

Due to questions about whether MM’s delirium was due to baclofen withdrawal versus intoxication, MM only received symptomatic management for her perceptual and behavioral disturbances throughout her stay. If this had been identified earlier, MM may have, instead, been restarted on a low-dose baclofen taper, which has been previously documented and recommended as a therapeutic approach [2-6]. In a review of 23 case reports of baclofen withdrawal, Leo and Baer (2005) found that 10 cases (43.5%) were managed by reinstituting baclofen alone, whereas reinstitution of baclofen with another agent, such as an antipsychotic, a benzodiazepine, or an anticonvulsant, were reported in 4 (17.4%), 6 (26.1%), and 1 (4.3%) case(s), respectively [4].

The presentation of baclofen withdrawal consists of a cluster of signs and symptoms, including perceptual disturbances, sleep disturbance, impaired thought processes, expansive affect, abnormal movements, disorganized speech, impaired attention, and disorientation, which may be variably present. In a patient presenting with these symptoms, it is imperative to rule out medical and pharmacological causes, especially in a patient with no history significant for psychosis or mania. While the majority of published case reports have described patients presenting in delirium from withdrawal from intrathecal baclofen, delirium from oral baclofen withdrawal can also be observed, as was the case with MM. Prompt identification of the etiology of this presentation will help guide subsequent management considerations by providers.

## Conclusions

Like withdrawal from intrathecal baclofen, withdrawal from oral baclofen can present with various cognitive, behavioral, perceptual, and motor disturbances. While symptomatic management of withdrawal symptoms with antipsychotics and benzodiazepines has been demonstrated, reinstituting and slowly tapering off baclofen has been demonstrated to be more targeted approach and effective approach.
